# Genome-Wide Gene Expression Profiling of Fertilization Competent Mycelium in Opposite Mating Types in the Heterothallic Fungus *Podospora anserina*

**DOI:** 10.1371/journal.pone.0021476

**Published:** 2011-06-28

**Authors:** Frédérique Bidard, Jinane Aït Benkhali, Evelyne Coppin, Sandrine Imbeaud, Pierre Grognet, Hervé Delacroix, Robert Debuchy

**Affiliations:** 1 Univ Paris-Sud, Institut de Génétique et Microbiologie UMR8621, Orsay, France; 2 CNRS, Institut de Génétique et Microbiologie UMR8621, Orsay, France; 3 CNRS, Centre de Génétique Moléculaire FRE3144, GODMAP, Gif sur Yvette, France; 4 UFR des Sciences du Vivant, Université Paris 7-Denis Diderot, Paris, France; 5 Univ Paris-Sud, Orsay, France; University College London, United Kingdom

## Abstract

**Background:**

Mating-type loci in yeasts and ascomycotan filamentous fungi (Pezizomycotina) encode master transcriptional factors that play a critical role in sexual development. Genome-wide analyses of mating-type-specification circuits and mating-type target genes are available in *Saccharomyces cerevisiae* and *Schizosaccharomyces pombe*; however, no such analyses have been performed in heterothallic (self-incompatible) Pezizomycotina. The heterothallic fungus *Podospora anserina* serves as a model for understanding the basic features of mating-type control. Its *mat+* and *mat−* mating types are determined by dissimilar allelic sequences. The *mat−* sequence contains three genes, designated *FMR1*, *SMR1* and *SMR2*, while the *mat+* sequence contains one gene, *FPR1*. *FMR1* and *FPR1* are the major regulators of fertilization, and this study presents a genome-wide view of their target genes and analyzes their target gene regulation.

**Methodology/Principal Findings:**

The transcriptomic profiles of the *mat+* and *mat−* strains revealed 157 differentially transcribed genes, and transcriptomic analysis of *fmr1^−^* and *fpr1^−^* mutant strains was used to determine the regulatory actions exerted by FMR1 and FPR1 on these differentially transcribed genes. All possible combinations of transcription repression and/or activation by FMR1 and/or FPR1 were observed. Furthermore, 10 additional mating-type target genes were identified that were up- or down-regulated to the same level in *mat+* and *mat−* strains. Of the 167 genes identified, 32 genes were selected for deletion, which resulted in the identification of two genes essential for the sexual cycle. Interspecies comparisons of mating-type target genes revealed significant numbers of orthologous pairs, although transcriptional profiles were not conserved between species.

**Conclusions/Significance:**

This study represents the first comprehensive genome-wide analysis of mating-type direct and indirect target genes in a heterothallic filamentous fungus. Mating-type transcription factors have many more target genes than are found in yeasts and exert a much greater diversity of regulatory actions on target genes, most of which are not directly related to mating.

## Introduction

Sexual reproduction is driven by specific gene expression programs required for identifying a sexually compatible partner, mating, meiosis and generation of progeny. Regulation of the genes involved in the identification of the mating partner and mating is well understood at a genome-wide level in the budding yeast *Saccharomyces cerevisiae* [1] and the fission yeast *Schizosaccharomyces pombe* [2], and these analyses have identified a limited number of cell specific-genes, most of which have a known function. Filamentous fungi provide an opportunity to study mating-type evolution and function in multicellular organisms, which is not possible in unicellular yeasts; however, no complete description of the regulation exerted by mating-type regulatory genes is available in the filamentous Ascomycetes. Furthermore, our understanding of the functions controlled by the mating-type transcription factors is very incomplete as most of their target genes remain unknown.

To help clarify the role of mating types, sexual development is being studied in an increasing number of filamentous fungi, such as *Podospora anserina* (reviewed in [3]), *Neurospora crassa* [4], [5], [6], *Sordaria macrospora* [7], *Gibberella zeae* (anamorph *Fusarium graminearum*) [8], [9], *G. moniliformis* (anamorph *F. verticillioides*) [10], *Aspergillus nidulans* [11], *Cochliobolus heterostrophus* (reviewed in [3]) and *Tuber melanosporum* [12]. In contrast to the yeasts, Pezizomycotina develop complex female organs and most heterothallic (self-incompatible) fungi also produce male cells, which can fertilize the female organs of the opposite mating type. This fertilization event relies on pheromone receptor systems, which resemble the archetypal system of peptidic pheromones found in the budding yeast [13], [14], [15], [16], [17], [18]. After fertilization, the female organ undergoes a series of complex differentiation events leading to the formation of several hundred asci, each one resulting from an independent meiotic event [19].

Sexual compatibility in heterothallic filamentous fungi is controlled by a single mating-type locus with two dissimilar allelic sequences, also termed idiomorphs [20]. One idiomorph is characterized by the presence of a gene encoding a transcription factor with a MATα_HMG domain [21], which was initially identified in the MATα1p protein of *S. cerevisiae* [22]. This gene is called *MAT1-1-1* in the standard nomenclature [23] and defines the *MAT1-1* idiomorph. The other idiomorph, *MAT1-2*, is characterized by the presence of a *MAT1-2-1* gene which encodes a transcription factor with a MATA_HMG domain. MAT1-1-1 and MAT1-2-1 are essential for fertilization in heterothallic Pezizomycotina [10], [24], [25], [26], [27] and development of the fruiting body [28], [29], [30], [31], [32].

Various other idiomorphic genes have been described in Pezizomycotina (see [33] for a review), notably *MAT1-1-2* and *MAT1-1-3*. MAT1-1-2 proteins contain a PPF domain, which is characterized by the conservation of three invariant residues, two prolines and one phenylalanine [34], while MAT1-1-3 transcription factors are characterized by an HMG-box and form a subgroup within MATA_HMG [21]. Their roles have been investigated in *P. anserina* [29], [32] and *N. crassa* [4], [35], [36], and they are required for post-fertilization development of the fruiting body. Despite numerous genetic analyses of mating-type gene functions, only a few mating-type target genes, essential for mating, have been identified. These genes are involved in the conserved pheromone/receptor system initially found in budding yeast.

**Figure 1:**
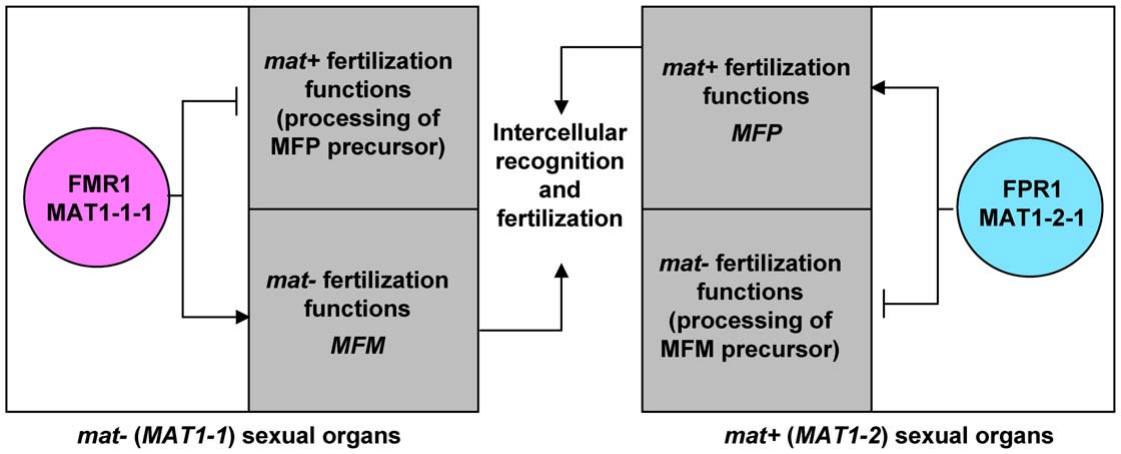
Regulation of fertilization by mating-type genes in *P. anserina*. Mating-type protein names are enclosed in colored circles: magenta, MATα-HMG protein; cyan, MATA-HMG proteins. Grey squares represent target genes present in *mat+* and *mat−* strains. The standard nomenclature is indicated below the *P. anserina*-specific gene names. *MFM* and *MFP* encode the pheromone precursors [13]. Arrows with heads and blunt ends indicate target-gene activation and repression, respectively. Data were compiled from [13], [28].

The control of cell-type specificity exerted by mating-type genes has been examined in detail through genetic approaches in the heterothallic filamentous ascomycete *P. anserina* (see Figure 1 for the correspondence of *P. anserina* and standard nomenclature for *MAT* genes). These studies revealed that the main regulators of fertilization, FMR1 and FPR1, have activator and repressor activities on the functions required for fertilization (Figure 1) ([13], [28] and reviewed in [3]). Two other genes, *SMR1* (*MAT1-1-2*) and *SMR2* (*MAT1-1-3*), are present in the *mat−* idiomorph. These two genes are not involved in the activation of the *mat−* functions required for fertilization, although the MATA_HMG transcription factor, SMR2, plays a minor role in the repression of the *mat+* fertilization functions in *mat−* strains. Subsequent studies have identified only two target genes of FMR1 and FPR1. These target genes encode the MFM and MFP pheromone precursors and are transcriptionally activated in a mating type-specific manner (Figure 1) [13]. Coppin *et al.* [13] have suggested that genes encoding pheromone precursor processing proteins are subjected to repression in a mating-type specific manner (Figure 1), but no such genes have been identified. The dual regulatory action of mating-type genes initially reported in *P. anserina* was also reported in *G. moniliformis* for target genes not directly involved in mating [10].

In this study, we present a transcriptomic genome-wide identification of the genes that are differentially transcribed between the *mat+* and *mat−* strains in a vegetative stage competent for fertilization. A total of 157 genes were identified and repression or activation of these genes was determined by analyzing the transcriptomic profile of *fpr1^−^* and *fmr1^−^* mutants. The data provided, for the first time, a complete description of the regulation exerted by mating-type genes on their target genes in a heterothallic Pezizomycotina. Many of the target genes are not involved in mating, in agreement with previous observations in *G. moniliformis* [10] and *S. macrospora* [7], [37], and deletion of 32 selected genes identified only two genes essential for mating. In addition, the search for common mating-type target genes in *P. anserina* and *G. moniliformis* or *S. macrospora* revealed statistically significant numbers of orthologous pairs; however, these conserved target genes have different transcriptional profiles.

## Results

### Time-course RT-qPCR analysis of mating-type gene transcript level during vegetative growth

*FMR1* and *FPR1* play essential roles in mating-type determination and fertilization (see Introduction). An RT-qPCR experiment was performed to determine the transcription pattern of these two genes and to identify the optimal time to search for differentially expressed genes in *mat−* and *mat+* mycelia. The transcript levels of mating-type genes were investigated on *mat+* and *mat−* mycelia harvested after incubation in Petri dishes for 24 h, 48 h, 72 h, 96 h and 120 h at 27°C under constant light. An additional experiment was performed with *mat−* and *mat+* strains fertilized by spermatization at 96 h and harvested 48 h later. At this time point, perithecia form ascogenous hyphae and nuclei undergo meiosis (Frédérique Bidard and Véronique Berteaux-Lecellier, unpublished results).

**Figure 2:**
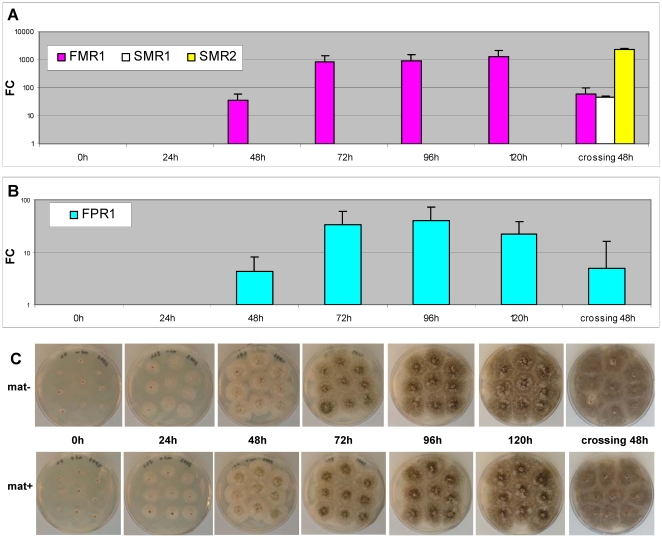
Time-course RT-qPCR analysis of mating-type gene transcript levels during vegetative growth and fruiting-body development. **A**: Relative quantification of *mat−* mating-type gene transcription using the Relative Expression Software Tool (REST) [69]. RNA was extracted from *mat−* colonies harvested during vegetative growth (24 h, 48 h, 72 h, 96 h and 120 h) and from *mat−* colonies grown for 48 h after fertilization with *mat+* microconidia (crossing 48 h). The reference time for *FMR1* fold-change determination was 24 h. The reference time for *SMR1* and *SMR2* fold-change determination was 120 h. Quantification cycle (Cq) values used for relative quantification are in Table S1. **B**: Relative quantification of *mat+* mating-type gene transcription with REST [69]. RNA was extracted from *mat+* colonies harvested during vegetative growth (24 h, 48 h, 72 h, 96 h and 120 h) and from *mat+* colonies grown for 48 h after fertilization with *mat−* microconidia (crossing 48 h). The reference time for *FPR1* fold-change determination was 24 h. Cq values used for relative quantification are shown in Table S1. **C**: Time course development of colonies. Upper row of Petri dishes: *mat+* strain. Bottom row of Petri dishes: *mat−* strain.

The quantification cycle (Cq) values for *FMR1* and *FPR1* cDNAs were very high at 24 h, indicating that their expression was very low at the beginning of vegetative growth (Table S1). The transcript levels of these two genes increased markedly at 48 h and reached a plateau at 72 h (Figure 2A and B). The nine colonies grown in a Petri dish made contact at 48 h (Figure 2C); thus, maximal *FMR1* and *FPR1* transcript levels occurred 24 h after confluence. A decrease in *FMR*1 and *FPR1* transcript levels was observed 48 h after fertilization.

Transcript levels of the *SMR1* and *SMR2* genes were undetectable throughout vegetative development until 120 h, at which time very low levels of transcripts were detectable in the *mat−* mycelium (Table S1). The transcript levels of these two genes increased strongly after fertilization (Figure 2A) in agreement with their essential roles during perithecium development [32], [38]. In line with our usual experimental conditions, fertilization was performed typically with 96 h-old mycelia, corresponding to the middle of the plateau phase of maximum transcript accumulation for *FMR1* and *FPR1*. This time-point was thus used in subsequent experiments to determine the genes that were differentially transcribed in *mat−* and *mat+* cultures.

### Transcriptomic profiling of *mat−* and *mat+* strains

A transcriptomic comparison of different mating-type strains provided an in-depth analysis of the genes that are differentially transcribed in the *mat−* and *mat+* strains. RNA was extracted from cultures grown under constant light for 96 h and prepared for hybridization (see Materials and Methods) to whole genome gene-expression microarrays developed for *P. anserina* [39].

Genes were defined as up-regulated in the *mat+* strain if their transcript levels showed a ≥2-fold change (FC≥2) with a p-value of <0.005 in the *mat+* strain compared to the *mat−* strain (*mat+ vs mat−* comparison). Reciprocally, genes were defined as up-regulated in the *mat−* strain when they showed a ≤2-fold change (FC≤−2) with a p-value of <0.005 in the *mat+ vs mat−* comparison. A total of 157 genes were differentially transcribed in the *mat+ vs mat−* comparison (Table S2); 88 genes accumulated more transcripts in *mat+* than in *mat−* strains (Table S3), and 69 genes accumulated more transcripts in *mat−* than in *mat+* strains (Table S4).

Next, we searched for genes expressed in a mating-type specific manner. Assuming that genes not expressed in one mating type should display a signal-to-standard-deviation ratio (SSR)≤3 [39], 13 genes were identified that were transcribed exclusively in the *mat+* strain and six genes were identified that were transcribed exclusively in the *mat−* strain (Table 1). Two of these genes have an ortholog with a known function in *S. cerevisiae*: *STE6*, the ortholog of Pa_5_11640 (ABC transporter), is involved in the transmembrane export of the lipophilic pheromones [40], and *FBP1*, the ortholog of Pa_4_9360 (fructose-1,6-bisphosphatase), which has no reported role in yeast mating. These 19 genes with mating type-specific expression did not include genes encoding the pheromone precursors and the pheromone receptors. This is in agreement with previous genetic analyses, which suggested that, although *mat+* and *mat−* pheromone genes are transcribed at low levels in *mat−* and *mat+* vegetative nuclei, respectively, tight repression of the expression of these genes in inappropriate cells takes place at the post–transcriptional level [13]. Interestingly, a low level of transcription was reported for an *N. crassa* pheromone receptor gene in inappropriate mating-type cells and a role in pheromone gene transcription was evidenced [41].

**Table 1:** Main features of genes specifically transcribed in *mat+* (FC≥2) or *mat−* (FC≤−2) strains.

Gene number^a^	FC	Gene name or function	Class	FPR1^b^	FMR1^c^
Pa_4_3858 (Δ)	48.16	unknown function	4	A	0
Pa_1_24410 (Δ)	27.87	SAM-dependent methyltransferase	4	A	0
Pa_5_3435 (Δ)	7.76	unknown function	4	A	0
Pa_5_9770 (Δ)	5.96	Asp protease	4	A	0
Pa_2_13290	3.42	unknown function	4	A	0
Pa_1_16335	3.28	unknown function	5	0	R
Pa_4_9360	2,67	fructose-1,6-bisphosphatase	8	0	0
Pa_2_8800	2,64	unknown function	4	A	0
Pa_6_9980	2,53	unknown function	8	A	0
Pa_5_11640	2,34	ABC transporter	8	0	0
Pa_7_2870	2,31	unknown function	4	A	0
Pa_1_20510	2,05	sugar transporter	2	A	R
Pa_2_7590	2,02	unknown function	8	0	0
Pa_3_10430	−2,08	unknown function	8	0	0
Pa_1_6014	−2,62	unknown function	1	0	0
Pa_4_1433	−2,64	unknown function	8	0	0
Pa_3_10350	−3,61	unknown function	8	0	0
Pa_6_10330 (Δ)	−3,91	polyketide synthase	5	0	A
Pa_4_1290	−5,05	carbohydrate esterase	5	0	A

a Genes selected for deletion are marked with Δ.

b A: gene induced by FPR1; R: gene repressed by FPR1; 0: gene not controlled by FPR1.

c A: gene induced by FMR1; R: gene repressed by FMR1; 0: gene not controlled by FMR1.

The consistency of microarray data with RT-qPCR analyses was tested for 14 genes selected to cover a wide range of FC values. Eleven genes displayed a similar FC direction in both experiments (Table S5). As commonly observed in the validation of array data, the FC values were much greater in the RT-qPCR than in the microarray experiments for the genes that were induced strongly [42], [43]. The strongest differences were observed for the *MFM* and *MFP* transcripts, which yielded dramatically low Cq values in the *mat−* and *mat+* strains, respectively (Table S6), suggesting that these two genes may be among those with the highest transcription level at this stage of development. Similarly, the pheromone precursor gene of *N. crassa*, *mfa-1*, was identified as the most abundant clone in starved mycelial cDNA libraries [18]. Of the 14 genes selected for microarray validation, three genes had an FC value close to one with non-significant p-values; microarray experiments gave typically more reliable results than RT-qPCR for genes that displayed low induction levels.

### Transcriptomic profiling of *fmr1* ^−^ and *fpr1* ^−^ mutant strains

Two types of genetic differences can account for differential transcription in *mat+* and *mat−* strains. The first type includes the two mating-type genes that are expressed at 96 h, namely *FPR1* in the *mat+* strain and *FMR1* in the *mat−* strain. Another possible type of genetic difference is the single nucleotide polymorphism (SNP) between *mat+* and *mat−* strains. In these isogenic strains, the polymorphism is restricted to the environment of the mating-type locus and may be associated with differential gene transcription in *mat+* and *mat−* strains. The effect of FMR1 and FPR1 on the transcription of the 157 genes, differentially transcribed in *mat+* and *mat−* strains, was determined by transcriptomic profiling of the *fmr1^−^* and *fpr1^−^* strains [44]. The *mat− vs fmr1^−^* and *mat+ vs fpr1^−^* comparisons (Table S2) enabled the identification of the target genes (direct or indirect) of FMR1 and FPR1, respectively. Genes that were differentially transcribed due to mating-type linked SNPs were differentially transcribed in the *fpr1^−^vs fmr1^−^* comparison (Table S2). Taken together, these comparisons allowed us to determine the contribution of FMR1, FPR1 and SNPs towards the transcription of each gene differentially expressed in *mat+* and *mat−* strains.

**Figure 3:**
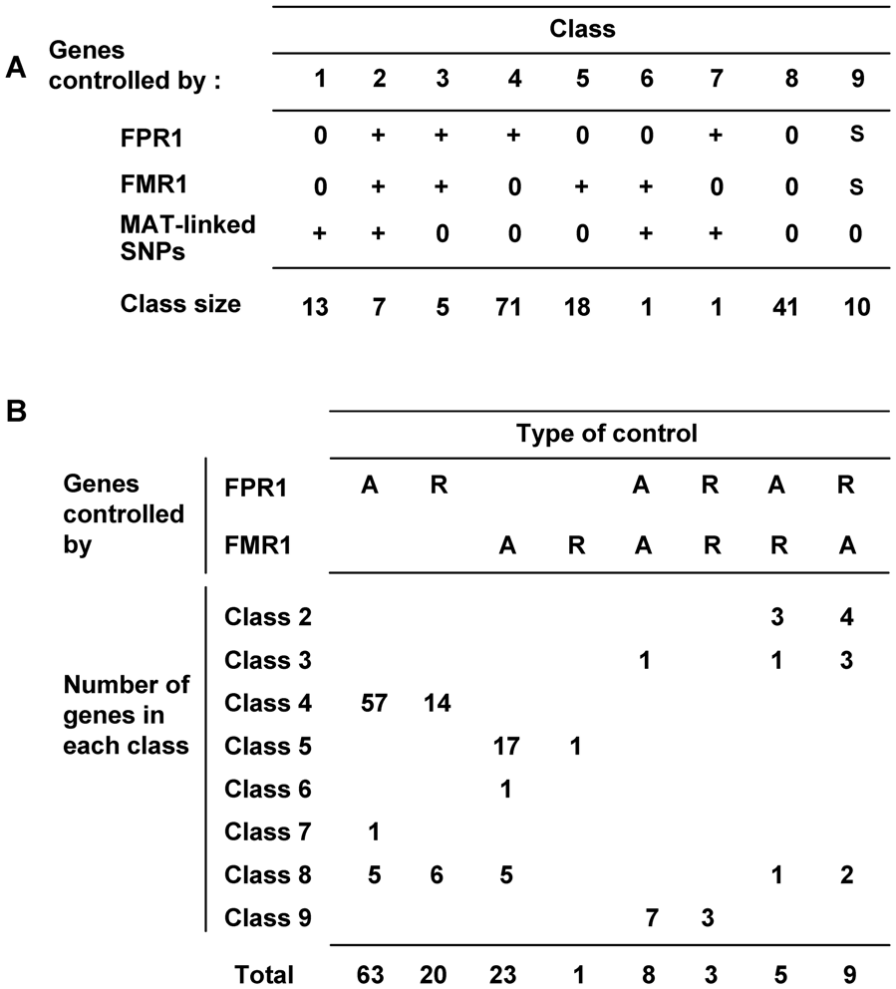
Partitioning of *mat+ vs mat−* differentially transcribed genes. **A**: Partitioning of differentially transcribed genes into classes controlled by mating-type genes or mating-type linked SNPs. Genes with FC≥2 or FC≤−2 (P<0.005) in *mat+ vs fpr1^−^*, *mat− vs fmr1^−^* and *fpr1^−^ vs fmr1^−^* comparisons are considered as being controlled by FPR1, FMR1 and mating-type linked SNPs, respectively. 0: no regulation; +:induction or repression; s: similar control in *mat+* and *mat−* strains. **B**: Partitioning of the mating-type target genes into groups with identical transcriptional patterns. Genes with FC≥2 in *mat+ vs fpr1^−^* and *mat− vs fmr1^−^* comparisons were considered as activated (A) by FPR1 and FMR1, respectively. Genes with FC≤−2 in *mat+ vs fpr1^−^* and *mat− vs fmr1^−^* comparisons were considered as repressed (R) by FPR1 and FMR1, respectively.

The 157 genes differentially transcribed in the *mat+ vs mat−* strains were divided into eight classes based on their transcription patterns (Table S7), and the regulatory effect of FMR1 or FPR1 (activation or repression) on each target gene was inferred from the FC values. It should be noted that the technology used in this study did not enable us to determine whether FMR1 and FPR1 were direct or indirect regulators of their target genes. The number of genes in each class and the control exerted by the mating-type genes are summarized in Figure 3.

Class 1 contained 13 genes differentially transcribed in *mat+* and *mat−* strains as a consequence of the mating-type linked SNPs and independent of any control by mating-type proteins. This class included the *FPR1* (Pa_1_20590) and *FMR1* genes. These two genes were not differentially transcribed in the *mat+ vs fpr1^−^* and *mat− vs fmr1^−^* comparisons, indicating that their transcription was not under the positive or negative control of the proteins that they encode. Classes 2 to 7 displayed all possible combinations of control by FMR1, FPR1 and mating-type linked SNPs. Strikingly, class 2 contained genes, which were differentially transcribed in *mat+* and *mat−* strains due to the effects of the three factors: FPR1 (in *mat+* strain), FMR1 (in *mat−* strain), and mating-type linked SNPs (in either *mat+* or *mat−* strains). Class 8 contained 41 genes differentially transcribed in *mat+* and *mat−* strains, but their control by FMR1, FPR1 or mating-type linked SNPs could not be clearly established because their FC scores or p-values were outside the selected significant values. A total of nine genes had FC scores close to the cut-off values (−2<FC<−1.8 or 1.8<FC<2) in the *mat+ vs fpr1^−^* comparison, as exemplified by Pa_4_5280 (FC = −1.97, P = 0.00048). The highly significant p-values associated with these FCs indicated that these genes were controlled by FPR1. Similarly, five genes appeared to be under the control of FMR1, based on their FCs and significant p-values in the *mat− vs fmr1^−^* comparison (*e.g.* Pa_1_3100, FC = 1.97, P = 0.00056). The analysis of FC values facilitated the identification of five additional genes from class 8, which were controlled by both mating-type genes. For each of these genes, FCs of wild-type-to-mutant comparisons were below the cut-off value, but their combination resulted in an FC value that corresponded to that observed in the *mat+ vs mat−* comparison. This was exemplified by Pa_7_9690 (protein-S-isoprenylcysteine O-methyltransferase), activated by FPR1 (FC = 1.69 in the *mat+ vs fpr1^−^* comparison) and repressed by FMR1 (FC = −1.75 in the *mat− vs fmr1^−^* comparison). Activation by FPR1 and repression by FMR1 were combined in the *mat+ vs mat−* comparison, yielding a 2.95-fold change. Following the same rationale, two genes (Pa_4_4560 and Pa_6_6730) were repressed by FPR1 and activated by FMR1, and two genes (Pa_4_3860 and Pa_2_11120) were activated by FPR1 and induced by SNPs linked to the *mat+* mating type. None of the remaining genes in class 8 displayed differential transcription in the *mat+ vs mat−* comparison, and FCs close to a value of one with significant p-values in the other comparisons. This precluded the existence of an undetermined regulatory factor that controls class 8 genes in addition to mating-type genes and SNPs. An additional ninth class (Table S7 and Figure 3) contained mating-type target genes that were not differentially transcribed in the *mat+ vs mat−* comparison (*e.g.* Pa_5_4645, FC = 1.33) because they were activated or repressed to a similar level in *mat+* and *mat−* strains (*e.g.* Pa_5_4645, FC = 3.46 in the *mat+ vs fpr1^−^* comparison and FC = 3.4 in the *mat− vs fmr1^−^* comparison). This class contained 10 genes with diverse functions (Table 2). Among the 167 genes examined in total, 132 genes were ultimately controlled by FMR1 or FPR1 or by both transcription factors, as shown in Figure 3B. The remaining 35 genes (13 genes from class 1 and 22 genes from class 8) were not included in this clustering. Class 1 consisted of genes exclusively under the control of mating-type linked SNPs, while the remaining genes from class 8 had FC values below the cut-off or not significant p-values and could not be included, with confidence, as genes controlled by FMR1, FPR1 or mating-type linked SNPs.

**Table 2:** Main features of genes up- or down-regulated to a similar level in *mat+* and *mat−* strains.

Gene number	FC^a^	FC^b^	Gene name or function	Class	FPR1^c^	FMR1^d^
Pa_2_4890	3.93	3.61	taurine catabolism dioxygenase	9	A	A
Pa_5_4645	3.46	3.4	NUDIX domain-containing protein	9	A	A
Pa_5_11800	3.08	2.85	MFS transporter	9	A	A
Pa_2_610	2.52	2.28	geranylgeranyl diphosphate synthase	9	A	A
Pa_0_495	2.33	2.05	unkown function	9	A	A
Pa_2_4855	2.08	2.39	unkown function	9	A	A
Pa_4_4010	2.07	2.39	nucleoside hydrolase	9	A	A
Pa_5_6710	−2.28	−2.17	unkown function	9	R	R
Pa_7_10775	−2.27	−2.9	unkown function	9	R	R
Pa_6_6690	−2.2	−2.09	acetyl transferase	9	R	R

a FC values in *mat+ vs fpr1^−^* comparison.

b FC values in *mat− vs fmr1^−^* comparison.

c A: gene activated by FPR1; R: gene repressed by FPR1.

d A: gene activated by FMR1; R: gene repressed by FMR1.

**Figure 4:**
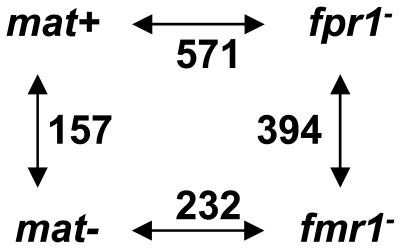
Number of differentially transcribed genes in wild-type and mutant strain comparisons. Cut-off values: FC≤−2 or FC≥2 and P<0.005.

The transcriptomic profiling of wild-type *vs* mutant strains revealed many more genes than the 157 that were differentially transcribed in the *mat+ vs mat−* comparison (Figure 4 and Table S2). The mutant strains may thus have accumulated adventitious mutations in addition to the *fpr1^−^* and *fmr1^−^* mutations. Ten genes were selected from the *fpr1^−^* and *fmr1^−^* strains to identify such putative mutations by sequencing (see Materials and Methods). No mutations were found in these ten genes, suggesting that their differential transcription might be the indirect result of adventitious mutations that have yet to be identified. The complete sequencing of the genome for the *fpr1^−^* and *fmr1^−^* strains is underway to identify these putative mutations.

### Functional annotation of mating-type regulated genes

The published functional annotation of the 157 genes, differentially transcribed in the *mat+* and *mat−* strains (class 1 to 8), and the 10 genes that were up- or down-regulated to similar levels in both strains (class 9) was completed for the proteins with unknown functions. Each of these protein sequences was searched against the non-redundant protein database at NCBI using BLASTP [45] and their putative functions were inferred from conserved domains (Table S8 and Table S9). The Functional Catalogue (FunCat) created by MIPS [46] was used to group the genes, differentially transcribed in *mat+* and *mat−* strains, according to their cellular or molecular functions (Table 3). Chi-squared tests indicated that the FunCat main functional categories were not differently distributed in the *mat+* and *mat−* strains. Approximately 50% of these proteins were of unknown function (FunCat class number 99). Among these proteins, eight did not produce a hit on any other protein and were specific to *P. anserina* (Table S8 and Table S9). Three genes (*nad1*, *2*, *3*) belonged to the mitochondrial genome and encoded the three sub-units of NADH dehydrogenase [47]. They were placed in class 1 (genes under the control of mating-type linked SNPs, Figure 3A and Table S7).

**Table 3:** Distribution of the 157 genes differentially transcribed in *mat+ vs mat−* in FunCat main functional categories.

FunCat main functional category ID	FunCat main functional category	Number of genes accumulating transcript in *mat−*	Number of genes accumulating transcript in *mat+*
Metabolism			
01	Metabolism	17	25
02	Energy	5	8
04	Storage protein		
Information pathways			
10	Cell cycle and DNA processing		
11	Transcription	1	4
12	Protein synthesis		
14	Protein fate (folding, modification, destination)	1	2
16	Protein with binding function	5	5
18	Protein activity regulation		1
Transport			
20	Cellular transport, transport facilitation and transport routes	6	9
Perception and response to stimuli			
30	Cellular communication/signal transduction mechanism		
32	Cell rescue, defense and virulence	6	2
34	Interaction with the environment	3	7
36	Systemic interaction with the environment		
38	Transposable elements, viral and plasmid proteins		
Developmental process			
40	Cell fate		
41	Development (systemic)		
42	Biogenesis of cellular components	1	3
43	Cell type differentiation		
45	Tissue-specific differentiation		
47	Organ differentation		
Localization			
70	Subcellular localization		1
73	Cell type localization		
75	Tissue localization		
77	Organ localization		
Unclassified			
98	Classification not yet clear-cut		
99	Unclassified proteins	37	45

The search for genes involved in the sexual process identified genes encoding the pheromones, their receptors and some genes required for pheromone biogenesis. Five of these genes were up-regulated in the *mat+* strain: the genes encoding the *mat+* lipophilic pheromone (*MFP*, Pa_2_2310) and the *mat−* pheromone receptor (*PRE2*, Pa_4_1380), and the orthologs of *RAM1* (farnesyl transferase β, Pa_4_7760), *STE24* (protein-S-isoprenylcysteine O-methyltransferase, Pa_7_9690) and *STE6* (ABC transporter, Pa_5_11640), which are required for the biogenesis of the lipophilic pheromone in *S. cerevisae* [48]. The first three genes clustered with the genes activated by FPR1 (class 4, Table S7), while the two last genes were activated by FPR1 and repressed by FMR1 (class 8, Table S7). The p-value for Pa_5_11640 (ABC transporter, STE6) was >0.005 but below the 0.05 upper limit of significance (class 8, Table S7). The prenylation of the *S. cerevisiae* lipophilic pheromone requires also the α subunit of farnesyl transferase, which is encoded by *RAM2* [49]. The ortholog of *RAM2* (Pa_2_9270) was not among the 157 genes that were differentially transcribed in *mat+* and *mat−* strains because its up-regulation in the *mat+* strain was below the cut-off value (FC = 1.59). However, the p-value for this gene was highly significant (P = 0.00001) (Table S2), suggesting that this gene may be considered as up-regulated in the *mat+* strain. Orthologs of the other genes required for lipophilic pheromone biogenesis were either not differentially regulated in *mat+* vs *mat−* strains (*RCE1*, *AFC1/STE24*, *STE14* and *STE23*) or were not found in the *P. anserina* genome (*AXL1*). Two genes involved in the sexual process were up-regulated in the *mat−* strain and were activated by FMR1: the genes encoding the *mat−* hydrophilic pheromone (*MFM*, Pa_1_8290) and the *mat+* pheromone receptor (*PRE1*, Pa_7_9070) (class 5, Table S7). Orthologs of the *KEX2* and *KEX1* genes, which are required for hydrophilic pheromone biogenesis in *S. cerevisiae* [48] were not differentially transcribed in the *mat+* and *mat−* strains. Pa_6_7350, the only protease-encoding gene to be up-regulated in the *mat−* strain, had no ortholog in *S. cerevisae* and no ortholog was found in *P. anserina* for the aminopeptidase *STE13*.

### Phenotypic analyses of target-gene deletions

The genes that displayed an FC value higher than 5 in the *mat+ vs mat−* comparison (Table S3) were selected for deletion and phenotype analysis, while two genes, Pa_2_2310 (*MFP*) and Pa_3_1710 (*AOX*), were deleted in previous studies [13], [50]. Fourteen additional genes from this list were selected based on (i) putative roles in reproduction (*e.g.* Pa_4_7760, farnesyltransferase β, RAM1), (ii) conservation in a wide range of fungi (*e.g.* Pa_2_7180), or (iii) functions of special interest (*e.g.*, the C6 transcription factors, Pa_2_6830 and Pa_6_3770). Nine additional candidates were selected among the genes that were up-regulated in the *mat−* strain (Table S4) based on similar criteria. The phenotype of the Pa_1_8290 (*MFM*) deletion was investigated in a previous study [13].

A total of 32 deletion strains were constructed and examined for male and female fertility and for possible defects in perithecium development (Table 4). The two mutant strains deleted for the *mat+* lipophilic pheromone receptor (PRE1, Pa_7_9070) and the *mat−* hydrophilic pheromone receptor (PRE2, Pa_4_1380) showed defects in sexual reproduction. The loss of the receptor for the *mat+* lipophilic pheromone resulted in female sterility in *mat−* strains, while *ΔPRE1 mat+* strains displayed wild-type fertility. Identical phenotypes were reported in *N. crassa* strains that were deleted for the lipophilic pheromone receptor, *pre-1* [41]. The *PRE2* deletion resulted in reciprocal phenotypes, namely female sterility in *mat+* strains, while *mat−* strains were not affected. The deletion of Pa_4_7760 (farnesyl transferase β, RAM1) was lethal, in contrast to the disruption of its ortholog in *S. cerevisiae* [49]. Farnesylation of Ras proteins is required for essential Ras function, suggesting that *RAM1* is an essential gene. Viable *RAM1* loss-of-function mutations in yeast have been attributed to an alternative prenylation process involving Cdc43p [49]. Similar to *P. anserina*, the deletion of *RAM1* is lethal in *Cryptococcus neoformans* [51], indicating that the farnesylation of Ras proteins cannot be compensated for by alternative pathways in these two fungi, although both contain orthologs of Cdc43p (Pa_5_4440 in *P. anserina*, CNAG_02756 in *C. neoformans*). The 29 remaining deletion strains were not impaired for mating and showed no defects in perithecium development. The mutant strains were also tested for their sensitivity to several inhibitors (see Materials and Methods) without revealing any differences between the mutant and the wild-type strains.

**Table 4:** Genes selected for deletion and phenotype of mutant strains.

Gene number	FC^a^	Gene name or function	mat specific expression	mutant phenotype
Pa_4_3858	48.16	unknown function	+	normal mating
Pa_1_24410	27.87	SAM-dependent methyltransferase	+	normal mating
Pa_4_1380	11.23	*PRE2*		*mat+* strains female sterile, *mat−* strains female fertile
Pa_5_3435	7.76	unknown function	+	normal mating
Pa_7_4100	7.5	unknown function		normal mating
Pa_3_3210	7.2	unknown function		normal mating
Pa_5_9770	5.96	Asp protease	+	normal mating
Pa_5_6960	5.94	unknown function		normal mating
Pa_1_9625	5.08	unknown function		normal mating
Pa_1_540	3.6	unknown function		normal mating
Pa_4_7760	3,03	farnesyltransferase subunit beta		essential
Pa_7_2860	2,5	cyclic-nucleotide phosphodiesterase		normal mating
Pa_2_9145	2,46	unknown function		normal mating
Pa_2_7180	2,39	unknown function		normal mating
Pa_2_9500	2,36	unknown function		normal mating
Pa_5_9790	2,35	unknown function		normal mating
Pa_1_5530	2,27	unknown function		normal mating
Pa_2_6830	2,26	C6 transcription factor		normal mating
Pa_2_3690	2,22	unknown function		normal mating
Pa_4_5450	2,17	unknown function		normal mating
Pa_4_9520	2,16	copper fist DNA binding domain protein		normal mating
Pa_2_1170	2,09	unknown function		normal mating
Pa_6_3770	2,06	C6 transcription factor		normal mating
Pa_6_7350	−6,56	protease		normal mating
Pa_7_9070	−5,62	*PRE1*		*mat+* strains female fertile, *mat−* strains female sterile
Pa_5_10930	−4,72	unknown function		normal mating
Pa_5_10935	−4,21	unknown function		normal mating
Pa_6_10330	−3,91	polyketide synthase	+	normal mating
Pa_5_10945	−3,27	unknown function		normal mating
Pa_7_875	−3,11	unknown function		normal mating
Pa_5_10940	−2,61	unknown function		normal mating
Pa_4_110	−2	unknown function		normal mating

a FC values in *mat+ vs mat−* comparison.

### Interspecies comparison of mating-type target gene transcriptional profiles

A cDNA macroarray analysis was conducted in *G. moniliformis* to compare a strain in which the *MAT1-2-1* gene was deleted with the *MAT1-2* wild-type strain [10]. The mutant and wild-type strains used in these experiments were the counterparts of those used in the *mat+ vs fpr1^−^* comparison, thus providing the opportunity to search for conserved mating-type target genes in these two heterothallic species. A total of 29 orthologous pairs were found in *G. moniliformis* MAT1-2-1 and *P. anserina* FPR1 target genes (P<0.0001) (Table 5 and Table S10), none of which encoded a protein with a known function directly related to mating. The *P. anserina* ortholog of clone #263, PaMpk3 (Pa_1_23930), is involved in osmotic stress resistance, but has no function in sexual development (Hervé Lalucque, Fabienne Malagnac and Philippe Silar, personal communication). The cDNA macroarrays did not provide an exhaustive representation of the genome, which possibly explains the low number of orthologous pairs identified as well as the absence of the expected pheromone and receptor pheromone gene pairs. Of the 29 genes regulated by MAT1-2-1 and FPR1 in *G. moniliformis* and *P. anserina*, none was repressed by both transcription factors, only 12 were activated by both transcription factors and a total of 17 genes were activated by MAT1-2-1 and repressed by FPR1 or *vice versa*, suggesting low conservation of the transcriptional profiles in *P. anserina* and *G. moniliformis*. Further correlation analysis is precluded because specific FC values were not available for *G. moniliformis* target genes.

**Table 5:** Cross-species searches of mating-type target genes.

Group 1 species	Group 1 transcription database (number of genes)	Group 2 species	Group 2 transcription database (number of genes)	Ortholog pairs	P value^a^	FC correlation (r)	P value^b^
*G. moniliformis*	*MAT1-2-1 vs ΔMT1-2-1* (160)	*P. anserina*	*FPR1 vs fpr1^−^* (571)	29	<0.0001	N/A^c^	N/A^c^
*S. macrospora*	*Smta-1 vs ΔSmta-1* (90)	*P. anserina*	*FPR1 vs fpr1^−^* (571)	16	<0.0001	0.27	0.30^d^
*S. macrospora*	*SmtA-1 vs ΔSmtA-1* (779)	*P. anserina*	*FMR1 vs fmr1* ^−^(232)	57	<0.0001	−0.25	0.06^e^

a p-value obtained from Chi-square analysis of interspecies orthologous pair distribution.

b p–value of interspecies FC correlation analysis.

c not applicable because specific FC values were not available.

d SpearmanRank Correlation: p-value = 0.29; Kendall tau Rank Correlation: p-value = 0.39.

e SpearmanRank Correlation: p-value = 0.70; Kendall tau Rank Correlation: p-value = 0.65.

*S. macrospora* is a homothallic species that contains a MATα_HMG gene (*SmtA-1*) and a MATA_HMG gene (*Smta-1*) homologous to *FMR1* and *FPR1*, respectively [52]. The SmtA-1 and Smta-1 target genes were identified in *S. macrospora*, using *N. crassa* microarrays [7], [37] and a search of the common target genes of FPR1 and Smta-1 revealed 16 orthologous pairs (P<0.0001) (Table 5 and Table S11). A total of 57 orthologous pairs were also found when searching for common target genes of FMR1 and SmtA-1 (P<0.0001) (Table 5 and Table S12). Only one gene (NCU03727, *ham-2*), the ortholog of Pa_ 2_9440, was reported to be involved in sexual reproduction in *N. crassa* [53]. The *ham-2* mutants are unable to differentiate female organs but male fertility is not affected. The transcriptional profiles of each group of orthologous genes were compared as described in [54]. The calculation of correlation based on the FC values of orthologous pairs reveal no shared transcriptional patterns for FPR1/Smta-1, nor for FMR1/SmtA-1 target genes (Table 5).

## Discussion

### Mating-type transcription factors FMR1 and FPR1 control a large number of target genes not directly involved in mating

**Figure 5:**
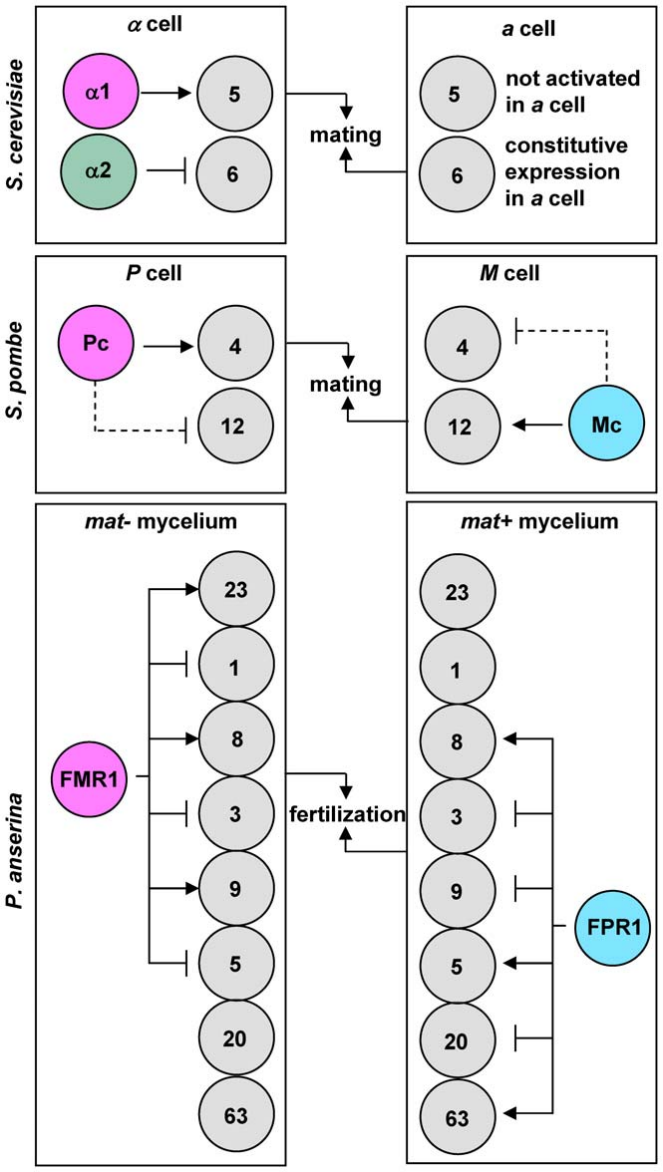
Cell-type regulation and target-gene numbers in *S. cerevisiae*, *S. pombe* and *P. anserina*. Mating-type protein names are enclosed in colored circles: magenta, MATα-HMG proteins; cyan, MATA-HMG proteins; green: homeodomain protein. Grey circles indicate different types of target genes and enclosed figures indicate their number. Mating-type genes are specific to each cell type, whereas target genes are present in both cell types. Arrows with heads and blunt ends indicate target gene activation and repression, respectively, except for ***a*** cells from *S. cerevisae*, which have specific regulation. Stipple arrows indicate hypothetical regulation. *S. cerevisiae* and *S. pombe* data were from compiled from [1], [2].

The fertilization-competent mycelium analysis described in this study provides the first global view of differential gene expression in strains of opposite mating type in Pezizomycotina. These analyses revealed that the sexual differentiation correlates with the differential transcription of many genes which are not directly involved in sexual reproduction. Genome-wide transcriptomic analyses were conducted in *S. cerevisiae* and *S. pombe* to determine differentially transcribed genes in cells of different mating types. Six **a**-specific genes and five α-specific genes were identified in *S. cerevisiae* [1] (Figure 5) and with the possible exception of one α-specific gene, all these genes are directly involved in some aspect of mating. In *S. pombe*, Mata and Bähler identified 12 M-specific genes and 4 P-specific genes [2] (Figure 5). Three of these cell type-specific genes were not essential for mating. In *P. anserina*, 157 genes were identified that were differentially transcribed in *mat+ vs mat−* strains and 10 genes were identified that were up- or down-regulated to a similar level in both strains. Thus, in *P. anserina*, the number of mating-type controlled genes (167) greatly outnumbers that in yeasts. In contrast with the cell-type specific genes in fission and budding yeasts, many *P. anserina* mating-type target genes appeared to be involved in a variety of biological processes, including general metabolism and energy production. A total of 32 genes were selected for deletion, including a few genes expressed in a mating-type specific manner. Only the genes encoding the pheromone receptors PRE1 (Pa_7_9070) and PRE2 (Pa_4_1380) were found to play a role in sexual reproduction, and one gene was found to be essential for viability (Pa_4_7760, farnesyl transferase β, RAM1). The deletion of the 29 other genes did not reveal any obvious defect in growth and/or mating. These 29 genes included three transcription factors (Pa_2_6830, Pa_4_9520 and Pa_6_3770), which were apparently unrelated to mating. Furthermore, ΔPa_2_6830 was genetically associated with either ΔPa_4_9520 or ΔPa_6_3770, and both double mutants behaved as the wild-type strain (data not shown). Although systematic deletions were not performed, these results suggested that the 167 mating-type target genes identified included very few candidates essential for fertilization or subsequent developmental steps. Currently, only pheromones [13] and pheromone receptor genes have been identified as mating-type target genes essential for mating in *P. anserina*.

Two hypotheses can account for the surprisingly high number of mating type-controlled genes not directly involved in mating. The first is based on the observation that *P. anserina* is a pseudo-homothallic (or secondary homothallic) species: it is encountered in nature as a self-fertile mycelium containing *mat+* and *mat−* nuclei. The optimal balance of the two types of nuclei for mycelium self-fertility may require that each type of nucleus controls complementary metabolic pathways. Although no differences in growth rate have been reported between homokaryotic and self-fertile mycelia in *P. anserina* in laboratory conditions, self-fertile mycelia may be favored in nature when less easily digestible nutrients are available. The second, and not exclusive, hypothesis is based on the idea that perithecium development relies on nutrients supplied by the mycelium [33], [55], [56]. The mating-type genes, which control perithecium development as well as mating, may be involved in the mobilization of nutrients necessary for the developing perithecia. In particular, the differences observed in the transcription profiles of *mat+* and *mat−* strains concern the metabolism of lipids whose role during sexual reproduction has been emphasized (reviewed in [57], [58]); for example, three genes encoding esterase-lipase are up-regulated in the *mat+* strain. Furthermore, six additional *mat+* up-regulated genes involved in lipid metabolism were identified, not including those necessary for processing of the lipophilic pheromone MFP. The lipid reserves may be metabolized during vegetative growth to release fatty acids and other compounds which could be used as nutrients to provide carbon skeletons and metabolic energy during sexual reproduction [56]. Although there are many obvious transcriptional differences between *mat+* and *mat−* strains, no conspicuous differences have ever been noticed during sexual reproductions, besides cell-type specificity. We cannot exclude that some differences do exist between *mat+* and *mat−* female strains during ascogonium formation and initial development after fertilization, although subsequent developmental stages in the maturing fruiting bodies converge in *mat+* and *mat−* female strains due to the complementary information brought by the male nucleus.

### Mating-type transcription factors FMR1 and FPR1 have a dual activating and repressing regulatory activity

Our analysis in *P. anserina* revealed a very complex regulatory pattern, which uses all eight possible combinations of regulation (activation and repression) by FMR1 and/or FPR1 (Figure 5). This pattern indicated that each mating-type protein has activator and repressor activity, and that either one or both mating-type proteins may control each target gene. The complex regulation exerted by mating-type genes has been anticipated by previous genetic analyses, which predicted that FPR1 acts as an activator for *mat+* function and as a repressor for *mat−* functions. FMR1 was predicted to have a symmetric but inverse activity to FPR1 (see Introduction and Figure 1). Subsequent genetic studies indicated that the repressor activity of each transcription factor may operate at the level of post-transcriptional pheromone maturation ([13] and reviewed in [3]). In agreement with this prediction, our study indicated that the protein-S-isoprenylcysteine O-methyltransferase (Pa_7_9690), which is required for the maturation of the lipophilic pheromone in yeast [48], is activated by FPR1 and repressed by FMR1 (see Results and Table S7, class 8). Pa_5_11640 (ABC transporter, STE6) could be a good candidate for a similar type of regulation, but this requires confirmation because the p-values were above the threshold. Unfortunately, this study did not succeed in identifying any gene that could be activated by FMR1 and repressed by FPR1 and was required for hydrophilic pheromone maturation.

The dual action (repression and activation) described in this study for the *P. anserina* mating-type genes is also conserved in budding yeast (Figure 5) (reviewed in [59]) and in *G. moniliformis* [10]. The fact that the Pc and Mc proteins of *S. pombe* have been reported to use only gene inductions [2] may be due to experimental bias. Mata and Bähler have compared the transcriptome of M and P vegetative cells which overexpress Ste11p relative to corresponding M and P control cells. This enabled the authors to identify activated target genes, but the experimental design could not reveal target genes that were tightly repressed in the control cells since these genes could not be repressed further in cells overexpressing Ste11p. It is therefore possible that M-specific genes are activated in M cells and repressed in P cells in *S. pombe*, whereas P-specific genes are subject to a symmetrical but reversed action in M cells (Figure 5). The dual action of mating-type genes (activation and repression) may be a conserved feature in Ascomycota, although both types of control are operated by two mating-type proteins in yeast and only one in *P. anserina* and *G. moniliformis*. Further analyses in *S. pombe* and dissection of mating-type regulatory circuits in other heterothallic Ascomycota will help to test this hypothesis.

### Interspecies comparisons of mating-type regulated genes revealed conserved genes but no shared transcriptional pattern

The search for common target genes of the mating-type transcription factors revealed a highly significant number of orthologous pairs in the *P. anserina*/*G. moniliformis* and *P. anserina*/*S. macrospora* comparisons. This result was unexpected for SMTA-1 and FMR1, because SMTA-1 is not essential for fertilization and fruiting-body development in *S. macrospora* [7], in contrast to FMR1 in *P. anserina* [24]. However, SMTA-1 is a positive regulator of pheromone gene transcription, although it is not essential for their expression [7]. This observation suggests that SMTA-1 may be involved in the control of the same genes as essential mating-type transcription factors. Orthologous pairs of genes, which are essential for mating, have typically similar expression patterns across species. For instance, the transcription of genes encoding lipophilic pheromones is activated by MATA_HMG transcription factor FPR1 in *P. anserina* and mat a-1 in *N. crassa* [17]. Similarly, the genes encoding hydrophilic pheromones are activated by MATα_HMG transcription factor FMR1 in *P. anserina* and mat A-1 in *N. crassa* [17]. In contrast, correlation analysis of FC values in orthologous pairs revealed that transcriptional profiles were not maintained among the numerous conserved mating-type target genes that were not involved in mating. One possible explanation to this paradoxical observation is that, for various combinations of genes, different transcriptional profiles might result in the same metabolic effect. This effect may be the mobilization of nutrients during the vegetative phase to fulfil the formation of the fruiting bodies, as proposed above. Another possible explanation is that there is a selection for the conservation of a binding site for a mating-type transcription factor at a conserved chromosomal locus, rather than selection for the function of the target genes. The inter-species variations of transcription patterns would thus reflect random variations in the expression of target genes. Conservation of these binding sites around diverse genes may be required for spatial and temporal sequestration of transcription factors, thereby contributing to the control of the expression of these regulatory proteins.

### Conclusions

Our study demonstrated that mating-type genes control many more target genes than those that are directly required for fertilization. Dissection of the regulatory circuits revealed all eight combinations that are possible with two transcription regulators exerting dual control (repression or induction). It must be noted that these experiments were conducted in homokaryotic strains. The control in a heterokaryotic *mat+/mat*− self-fertile strain cannot be inferred from our results, as competition of FMR1 and FPR1 for binding sites may impact the transcription of target genes under dual control. Moreover, variation in the nuclear balance during the development of the heterokaryotic mycelium [60] may result in an additional layer of complexity to the temporal expression of target genes. The role of the mating-type target genes in a biological process related to sexual reproduction is still elusive and its elucidation will require both biochemical analyses of the metabolic pathways involving these genes and ChIP experiments to determine if they are primary or secondary target genes of FMR1 and FPR1. Mating-type regulatory proteins also play essential roles in the development of the ascogenous hyphae in the fruiting body, in association with the other mating-type proteins (reviewed in [33]). Further investigations will be necessary to determine how the switch from vegetative to reproductive tissues modifies the set of target genes that are controlled by FMR1 and FPR1.

## Materials and Methods

### Strains

The genetic and biological features of *P. anserina* were first described by Rizet and Engelmann [61] and current culture techniques can be found at http://podospora.igmors.u-psud.fr/methods.php. The strains used in this study were all derived from the *S* strain [62], which was used to determine the *P. anserina* genome sequence [63]. The *fmr1^−^* and *fpr1^−^* mutant strains used for transcriptomic profiling have been described previously [44]. Successive nonsense mutations were introduced at the beginning of *FMR1* and *FPR1* coding sequences, resulting in loss-of-function mutations. Deletion of genes identified in transcriptomic profiling was performed by targeted gene replacement in a strain defective for Pa*KU70*, a gene necessary for the non-homologous end joining recombination [44].

### Preparation of biological samples for RNA extraction

#### Vegetative cultures

Petri dishes containing minimal medium were covered with a cellophane sheet (cat#1650193, Bio Rad, Hercules, USA) and were inoculated with nine implants from *P. anserina mat+* or *mat−* cultures. The dishes were placed at 27°C under constant light (0 h) and were removed from the incubation room at different culture time points (24 h, 48 h, 72 h, 96 h and 120 h). With a growth rate of 7 mm/day, the mycelium reached confluence at 48 h. Mycelia were harvested by scraping with a microscope coverglass and excess water was removed with Precision Wipes (Kimberly-Clark, Reigate, United Kingdom). The mycelium was then stored in aliquots of no more than 100 mg in liquid nitrogen until RNA extraction. Quantitative PCR experiments were performed with five *mat+* plates and five *mat−* plates for each time point. Transcriptomic profiling at 96 h was performed with four biological replicates for each time point, with each plate being considered as a biological replicate.

#### Fertilized cultures

Plates were prepared as indicated above, except that the medium was covered with cheesecloth (Sefar Nitex 03-48/31, Sefar, Heiden, Swiss) instead of cellophane, which was completely degraded by *P. anserina* after 120 h. Crossing was performed at 96 h by spermatization: each *mat+* plate was washed with 2 ml of water to harvest the *mat+* spermatia, and the suspension was collected and poured onto a *mat−* plate to fertilize the *mat−* female organs. After applying the *mat+* spermatia suspension onto the *mat−* plate, the suspension, which contained the *mat−* spermatia, was collected and poured back onto the *mat+* plate to fertilize the *mat+* female organs. Mycelium and perithecia were harvested by scraping the cheesecloth with a microscope coverglass. Quantitative PCR experiments were performed with five *mat+* plates and five *mat−* plates. Each plate was considered to be a biological replicate.

### RNA extraction

A maximum of 100 mg of mycelium was recovered from liquid nitrogen and ground in a Mikro-Dismembrator (Sartorius, Goettingen, Germany) for 1 min at 2600 rpm in vessels containing 9 mm chromium steel grinding balls frozen in liquid nitrogen. The resulting powder was suspended in RLT buffer (700 µl) (Qiagen, Hilden, Germany) and the suspension was passed through a Qiashreddrer column (Qiagen, Hilden, Germany). Total RNA was purified on RNeasy Plant Mini Kit columns (ref 74904, Qiagen, Hilden, Germany) following the manufacturer's protocol, which included DNase treatment. For microarray experiments, washing with RPE buffer (500 µl and 350 µl) was performed a further two times to improve RNA quality. The quantity and quality of the total RNA was determined using a NanoDrop ND-1000 spectrophotometer (Nanodrop Technologies, Wilmington, USA) and by electrophoresis on 1% agarose gel. For microarray experiments, a further control was performed on the Bioanalyzer 2100 system (Agilent, Santa Clara, USA) as described previously [64].

### RT-qPCR analyses

Total RNA was reverse-transcribed with Superscript III (Invitrogen, Carlsbad, USA) and oligo(dT)_20_ as recommended by the manufacturer. RT-qPCR amplification mixtures (10 µl) contained 1× FastStart Universal SYBR Green Master (Rox) (Roche Diagnostics, Mannheim, Germany), 2 µl of a 1/10 dilution of the reverse-transcription reaction and 500 nM of forward and reverse primers. Reactions were run on a CFX96 device (Bio Rad, Hercules, USA). The cycling conditions comprised 10 min polymerase activation at 95°C and 45 cycles at 95°C for 10 sec and 60°C for 30 sec with plate read, followed by a melting curve. Each plate contained cDNA samples representing all of the conditions examined for one gene, a standard curve of three serial dilutions points (1/10 dilutions) and a no-template control. All measures were performed with five biological replicates for each condition. For microarray validation, the biological replicates used for RT-qPCR were different from those used in microarray experiments. All of the PCR efficiencies were over 85%. At least one primer in each primer pair was positioned on two consecutive exons (Table S13), ensuring specific detection of cDNA with minimal interference by genomic DNA. *MFM* and *MFP* genes are intron-less genes and were detected with primers that do not discriminate cDNA from genomic DNA: therefore, the detection of their cDNA was not below the level of genomic DNA contamination. For these two genes, a non-reverse-transcribed control was performed on each biological replicate under the same conditions as the reverse-transcribed samples to determine the level of genomic DNA contamination. Measurements were considered as significant when the genomic contamination was five cycles above the cDNA signal.

For each RT-qPCR analysis (time course and microarray validation), eight housekeeping genes (Table S13) were screened with geNorm [65] to identify the best reference genes for each type of experiment. The minimum number of reference genes was selected that had a pairwise variation (V) below 0.15. If two genes were sufficient to reach a value of 0.15 for V, three reference genes were used according to MIQE guidelines [66], provided that the addition of the third reference gene did not increase V above 0.15. Pa_7_8490 (ubiquitin), Pa_4_8980 (TATA-box binding protein), Pa_3_5110 (glyceraldehyde phosphate dehydrogenase) [67] and Pa_5_5390 (histone H2A) were used for normalization of the time-course RT-qPCR (V = 0.15). Pa_7_8490 (ubiquitin), Pa_3_5110 (glyceraldehyde phosphate dehydrogenase) [67] and Pa_1_16650 (ribosomal protein AS1) [68] were used as reference genes for normalization of the microarray RT-qPCR validation (V = 0.031).

RT-qPCR normalization, standard error computation and statistical analyses were performed with REST-MCS and REST 2009 (Qiagen, Hilden, Germany) [69].

### Labelling of cDNA, hybridization and microarray analyses

The construction and optimization of gene expression microarrays for *P. anserina* has been described previously [39]. Briefly, the microarrays consisted of a 4×44 K platform (AMADID 018343, Agilent, Santa Clara, USA) containing 10,556 probes on each array with each probe present in four replicates.

For the transcriptome microarray experiments, target preparation, hybridization and washing were performed according to the two-color microarray-based gene expression analysis instructions (version 5.0, February 2007) as described by the manufacturer (Agilent, Santa Clara, USA). One-microgram aliquots of total RNA were amplified and Cy-labelled with Agilent's Low RNA input fluorescent linear amplification (LRILAK) PLUS kit and the Two-Color RNA Spike-in Kit (Agilent, Santa Clara, USA). The labelling efficiency and the product integrity were checked as described previously [64].

Four biological replicates labelled with Cy-3, for each of the different experimental conditions were compared with a common reference labelled with Cy-5, in indirect comparisons. The common reference was obtained by mixing RNA extracted from the different conditions as indicated in [39]. A total of 825 ng of each of the Cy3- and Cy5-labeled targets was mixed and incubated on microarray slides for 17 h at 65°C in a rotating oven (10 rpm) using an Agilent *in situ* hybridization kit (Agilent, Santa Clara, USA). The slides were washed and dried by centrifugation at 800 rpm for 1 min.

Microarrays were scanned using the Agilent DNA microarray Scanner (Agilent, Santa Clara, USA) at a resolution of 5 microns using the extended dynamic range (XDR) feature. Spot and background intensities were extracted with the Feature Extraction (FE, v9.5.3) software (Agilent, Santa Clara, USA) using the GE2-v4_95_Feb07 default protocol. Preliminary array quality was assessed through the use of Agilent control features as well as spike-in controls (Agilent 2-Color Spike-in Kit for RNA experiment). Subsequent flagging was done according to the GenePix Pro software (Molecular Devices Sunnyvale, USA) nomenclature, which included four flag levels (good [100], bad [−100], not found [−50], moderate [0]). FE-software normalized data (Lowess normalized, local background subtracted) were processed with MAnGO [70]. A moderated t test with adjustment of p-values [71] was computed to measure the significance of each difference of expression. Genes were considered as differentially transcribed if their transcript levels showed a ≥2-fold change (FC≥2) or ≤2-fold change (FC≤−2) with a p-value of <0.005.

### Microarray data accession number

All microarray data is MIAME compliant. The raw data has been deposited in the MIAME compliant Gene Expression Omnibus database [72] and is accessible through the GEO Series accession number GSE27297.

### Selection and sequencing of genes from *fpr1^−^* and *fmr1^−^* strains

Ten genes were selected in *fpr1^−^* and *fmr1^−^* strains to identify putative adventitious mutations that might have occurred during the introduction of site-directed mutations in *FPR1* and *FMR1* [44]. As the two mutant strains were generated independently, it is highly unlikely that they would harbor identical mutations. Therfore, it was assumed that an adventitious mutation in a given gene should affect its expression in either one of the two mutant strains, while its expression should be similar in the three other strains considered, namely the other mutant strain, the *mat+* and the *mat−* strains. Pa_1_23560, Pa_3_11210, Pa_5_4750, Pa_5_10440 and Pa_7_130 displayed differential transcription with highly significant p-value in the *fpr1^−^ vs fmr1^−^* and *fpr1^−^ vs mat+* comparisons but not in the *mat+ vs mat−* and *fmr1^−^ vs mat−* comparisons (Table S2); thus these genes were selected as the best putative targets for adventitious mutations in the *fpr1^−^* strain. Based on a similar rationale, Pa_1_970, Pa_1_16110, Pa_2__4340, Pa_4_4670 and Pa_6_6260 were selected as possible targets for adventitious mutations in the *fmr1^−^* strain. The selected genes were amplified from the appropriate DNA with Dream Taq (Fermentas, St Leon-Rot, Germany) in accordance with the manufacturer's instructions using primers located 1000 bp upstream and 1000 bp downstream of the translation initiation codon. Each amplification product was sequenced with upstream and downstream primers and primers targeted to regions around the initiation codon. Sequences were assembled with CAP3 [73], which is available on http://pbil.univ-lyon1.fr/cap3.php, and were compared with wild-type sequences using BLAST [45].

### Gene annotation and the search for orthology

Protein sequences were searched against the NCBI non-redundant protein sequence library using BLASTP [45]. FUNGIpath [74] was used to search for orthologous genes on http://embg.igmors.u-psud.fr/fungipath/.

### Gene deletion

The construction of deletion cassettes was based either on the *Asc*I/*Mlu*I method or on the *N. crassa* strategy for high-throughput generation of gene deletions [75].

The *Asc*I/*Mlu*I method is based on cloning an *Mlu*I fragment containing the resistance selection gene in an *Asc*I linearized plasmid containing the 3′ and 5′ flanking regions of the gene that was selected for deletion. This plasmid was obtained by the amplification of a vector containing the selected gene with primers targeted to the 5′ and 3′ borders of the coding sequence to be deleted and with their 3′ ends oriented towards the 5′ and 3′ untranslated regions. These diverging primers were designed with an *Asc*I site at their 5′ end in order to amplify the vector in a linearized form, ending with an *Asc*I site and lacking the coding sequence of the selected gene. The selection gene in an *Mlu*I fragment was obtained by amplification of the *hph* gene from the pBCHygM vector with the primer pair, 5_CPC1_Mlu (5′-gcgacgcgtccgagatgcgccgcgtgc-3′) and 3_Ttrpc_Mlu (5′-gtcacgcgtagaggatcctctagcta-3′), followed by digestion with *Mlu*I. The pBCHygM vector is a derivative of pBC-hygro [76], which has lost the *Mlu*I site in the trpC terminator.

The *N. crassa* strategy has been previously described [75] and included two modifications aimed at minimizing errors in the 5′ and 3′ flanking regions and the *hph* cassette obtained from pMOcosX [77]. First, the high-fidelity *Pfu* polymerase (Promega, Madison, USA) was used instead of the low-fidelity *LA Taq* polymerase (TaKaRa BIO Inc, Shiga, Japan) and the cycle conditions were adapted to minimize amplification errors. If amplifications with *Pfu* on 20 cycles failed, 10 additional cycles were performed, and if 30 cycles were not successful, the *LA Taq* polymerase was used according to the manufacturer's instructions. The second modification involved the addition of an A*sc*I site at each end of the deletion cassette. These sites were used to linearize and separate the deletion cassette from the vector prepared by amplification in *Escherichia coli* prior to the transformation of *P. anserina*.

Linearized DNA containing the 3′ and 5′ untranslated regions of the gene to be deleted and the *hph* selection gene [77], was used for the transformation of the *P. anserina* Δ*PaKU70* strain as described in http://podospora.igmors.u-psud.fr/methods.php#transfo. In each mutant strain, accurate gene replacement was checked by Southern blot analysis and one transformant containing the correct deletion was crossed with a wild-type strain to generate both *mat+* and *mat−* mutant strains lacking the Δ*PaKU70* deletion.

### Phenotype analyses

For each deletion, both *mat+* and *mat−* mutant strains were tested for fertility. The number of male cells active in the fertilization of a compatible wild-type tester strain (used as a female) was determined as previously described [13]. Crosses homozygous for each deletion were performed to search for possible defects in the development of the fertilized female organs. The capacity of *mat+*/*mat*− heterokaryotic deletion strains to grow and reproduce on different carbon sources was tested as described in [78]. Growth and fertility of *mat+*, *mat−* and *mat+*/*mat−* deletion strains were tested on minimal media containing inhibitors that act on different cell compartments: nikomycine (0.1 mg/ml), which acts on the cell wall by inhibiting chitin synthase; fludioxonil (0.01 mg/ml), which acts on osmoregulation; sodium dodecylsulfate (0.03%) which acts on the cell membrane; and caffeine which induces pleiotropic effects. The concentration of each inhibitor caused an approximately 50% decrease in the wild-type growth rate.

### Calculation of interspecies correlation

FUNGIpath [74] was used to identify systemically orthologous groups of genes for pairwise comparisons between *P. anserina* FPR1 and *G. moniliformis* MAT1-2-1 target genes (Table S10), *P. anserina* FPR1 and *S. macrospora* Smta-1 target genes (Table S11), and *P. anserina* FMR1 and *S. macrospora* SmtA-1 target genes (Table S12). The cut-off for selecting differentially transcribed genes in *P. anserina* comparisons was lowered to ≥1.5-fold change (FC≥1.5) and a ≤1.5-fold change (FC≤−1.5) with a p-value of <0.005. P-values for the overlap of gene sets between *P. anserina* and *G. moniliformis* or *S. macrospora* (Table 5) were determined from a 2×2 contingency table using a Chi-square test with Yates correction run on http://www.graphpad.com/quickcalcs/contingency1.cfm (GraphPad Software Inc, La Jolla, USA). The contingency table compared the number of orthologs (group 1, outcome 1) identified in the total number of target genes (group 1, outcome 2) of a given mating-type transcription factor (*G. moniliformis* MAT1-2-1, Smta-1 or SmtA-1) with the number of targets genes (group 2, outcome 1) identified for the corresponding transcription factor in the 10,556 genes of *P. anserina* (group 2, outcome 2). Calculation of interspecies correlation (Table 5) were performed as described in [54] from log-transformed FC values. Pearson correlations, p-values calculations from T-tests, Spearman rank correlations and Kendall's Tau were performed on http://www.wessa.net/stat.wasp [Wessa, P. (2011), Free Statistics Software, Office for Research Development and Education, version 1.1.23-r6].

## Supporting Information

Table S1 Time-course RT-qPCR analysis during vegetative growth. Cq values for mating-type gene and reference gene cDNAs are reported during vegetative growth (48 h, 72 h, 96 h and 120 h) and crossing (48 h). N/A: Cq value not available because out-of-scale.
(XLS)

Table S2 Whole genome transcriptomic comparisons of wild-type and mutant strains. Fold change (FC), adjusted p-value (p-value) and the binary logarithm of the arithmetic mean of compared intensities (A) are indicated for the 10,556 *P. anserina* genes and for each comparison (*mat+ vs mat−*, *fpr1^−^ vs fmr1^−^*, *mat+ vs fpr1^−^* and *mat− vs fmr1^−^*). Genes with FC values above cut-off and significant p-values are marked by “1” in the “selection” column for each comparison. FC cut-offs: FC≤−2 or FC≥2. p-value cut-off: P<0.005.
(XLS)

Table S3 Main features of genes accumulating transcripts in the *mat+* strain.
(DOC)

Table S4 Main features of genes accumulating transcripts in the *mat−* strains.
(DOC)

Table S5 RT-qPCR validation of microarray data. Cq values are in Table S6.
(DOC)

Table S6 RT-qPCR validation of microarray. Cq values for mating-type target gene and reference gene cDNAs are reported for *mat+* and *mat−* strains in 96 h-old mycelium.
(XLS)

Table S7 Partitioning of the *mat+ vs mat−* differentially transcribed genes according to their FC value in wild-type to mutant comparisons. Genes with positive FCs (FC≥2, P<0.005) in the *mat+ vs fpr1^−^* and *mat− vs fmr1^−^* comparisons were considered as being activated (A) by FPR1 and FMR1, respectively. Genes with negative FCs (FC≤−2, P<0.005) in the *mat+ vs fpr1^−^* and *mat− vs fmr1^−^* comparisons were considered as being repressed (R) by FPR1 and FMR1, respectively.
(XLS)

Table S8 FunCat classification of genes up-regulated in the *mat+ vs mat−* strain comparison.
(XLS)

Table S9 FunCat classification of genes up-regulated in the *mat− vs mat+* strain comparison.
(XLS)

Table S10 Orthologous pairs of *P. anserina* FPR1 and *G. moniliformis* MAT1-2-1 target genes.
(XLS)

Table S11 Orthologous pairs of *P. anserina* FPR1 and *S. macrospora* SMTa-1 target genes.
(XLS)

Table S12 Orthologous pairs in *P. anserina* FMR1 and *S. macrospora* SMTA-1 target genes.
(XLS)

Table S13 Oligonucleotide primers used for RT-qPCR.
(DOC)
