# Supplementary material for: Genome-Wide Gene Expression Profiling of Fertilization Competent Mycelium in Opposite Mating Types in the Heterothallic Fungus Podospora anserina
Source: PLoS One. 2011 Jun 28;6(6):e21476. doi: 10.1371/journal.pone.0021476 (PMC3125171; doi:10.1371/journal.pone.0021476)
Supplement: Table S3 — Main features of genes accumulating transcripts in the mat+ strain. (DOC) [file pone.0021476.s003.doc]

**Table S3.** Main features of genes accumulating transcripts in the *mat+* strain.

| Gene number^a^ | FC | Gene name or function | mat+ specific expression | Class | FPR1^b^ | FMR1^c^ |
| --- | --- | --- | --- | --- | --- | --- |
| Pa_2_2310 | 144.05 | *MFP* |  | 4 | A | 0 |
| Pa_4_3858 (Δ) | 48.16 | unknown function | + | 4 | A | 0 |
| Pa_1_24410 (Δ) | 27.87 | SAM-dependent methyltransferase | + | 4 | A | 0 |
| Pa_4_1380 (Δ) | 11.23 | *PRE2* |  | 4 | A | 0 |
| Pa_5_3435 (Δ) | 7.76 | unknown function | + | 4 | A | 0 |
| Pa_7_4100 (Δ) | 7.5 | unknown function |  | 4 | A | 0 |
| Pa_3_3210 (Δ) | 7.2 | unknown function |  | 4 | A | 0 |
| Pa_5_9770 (Δ) | 5.96 | Asp protease | + | 4 | A | 0 |
| Pa_5_6960 (Δ) | 5.94 | unknown function |  | 4 | A | 0 |
| Pa_1_9625 (Δ) | 5.08 | unknown function |  | 4 | A | 0 |
| Pa_3_1710 | 4.58 | *AOX* |  | 4 | A | 0 |
| Pa_5_12980 | 3.64 | unknown function |  | 4 | A | 0 |
| Pa_1_540 (Δ) | 3.6 | unknown function |  | 4 | A | 0 |
| Pa_4_3160 | 3.54 | phosphoenolpyruvate carboxykinase |  | 4 | A | 0 |
| Pa_6_515 | 3.52 | unknown function |  | 7 | A | 0 |
| Pa_1_18670 | 3.43 | unknown function |  | 1 | 0 | 0 |
| Pa_2_13290 | 3.42 | unknown function | + | 4 | A | 0 |
| Pa_1_16335 | 3.28 | unknown function | + | 5 | 0 | R |
| Pa_4_1370 | 3.06 | amidohydrolase |  | 4 | A | 0 |
| Pa_4_7760 (Δ) | 3,03 | farnesyltransferase subunit beta |  | 4 | A | 0 |
| Pa_7_9690 | 2,95 | protein-S-isoprenylcysteine O-methyltransferase |  | 8 | A | R |
| Pa_7_1820 | 2,79 | mitochondrial NADH-ubiquinone oxidoreductase 1 |  | 4 | A | 0 |
| Pa_1_20140 | 2,77 | unknown function |  | 8 | A | 0 |
| Pa_5_4585 | 2,73 | unknown function |  | 4 | A | 0 |
| Pa_4_9360 | 2,67 | fructose-1,6-bisphosphatase | + | 8 | 0 | 0 |
| Pa_2_8800 | 2,64 | unknown function | + | 4 | A | 0 |
| Pa_4_7450 | 2,62 | ketopantoate hydroxymethyltransferase |  | 4 | A | 0 |
| Pa_6_540 | 2,61 | unknown function |  | 8 | 0 | 0 |
| Pa_6_9760 | 2,56 | nonribosomal peptide synthetase |  | 4 | A | 0 |
| Pa_1_19170 | 2,54 | unknown function |  | 1 | 0 | 0 |
| Pa_6_9980 | 2,53 | unknown function | + | 8 | A | 0 |
| Pa_7_2860 (Δ) | 2,5 | cyclic-nucleotide phosphodiesterase |  | 4 | A | 0 |
| Pa_5_820 | 2,49 | unknown function |  | 1 | 0 | 0 |
| Pa_6_350 | 2,48 | plasma membrane proteolipid 3 |  | 4 | A | 0 |
| Pa_2_5500 | 2,48 | cation-transporting ATPase |  | 3 | A | R |
| Pa_2_9145 (Δ) | 2,46 | unknown function |  | 4 | A | 0 |
| Pa_5_7250 | 2,45 | unknown function |  | 4 | A | 0 |
| Pa_0_690 | 2,42 | acyl CoA thioesterase |  | 4 | A | 0 |
| Pa_2_7170 | 2,4 | glycosyltransferase |  | 4 | A | 0 |
| Pa_6_11620 | 2,4 | methionine permease |  | 4 | A | 0 |
| Pa_2_4810 | 2,4 | unknown function |  | 4 | A | 0 |
| Pa_2_7180 (Δ) | 2,39 | unknown function |  | 4 | A | 0 |
| Pa_5_9600 | 2,39 | isovaleryl-CoA dehydrogenase |  | 4 | A | 0 |
| Pa_4_80 | 2,37 | unknown function |  | 1 | 0 | 0 |
| Pa_5_12470 | 2,36 | unknown function |  | 1 | 0 | 0 |
| Pa_3_8590 | 2,36 | cytochrome c |  | 4 | A | 0 |
| Pa_2_9500 (Δ) | 2,36 | unknown function |  | 4 | A | 0 |
| Pa_1_30 | 2,35 | unknown function |  | 1 | 0 | 0 |
| Pa_5_9790 (Δ) | 2,35 | unknown function |  | 4 | A | 0 |
| Pa_5_11640 | 2,34 | ABC transporter | + | 8 | 0 | 0 |
| Pa_5_11460 | 2,32 | abhydrolase |  | 4 | A | 0 |
| Pa_7_2870 | 2,31 | unknown function | + | 4 | A | 0 |
| Pa_0_1270 | 2,29 | MSF superfamily |  | 3 | A | A |
| Pa_1_20590 | 2,27 | *FPR1* |  | 1 | 0 | 0 |
| Pa_4_860 | 2,27 | unknown function |  | 4 | A | 0 |
| Pa_1_5530 (Δ) | 2,27 | unknown function |  | 4 | A | 0 |
| Pa_2_6010 | 2,26 | cholesterol oxidase |  | 4 | A | 0 |
| Pa_2_6830 (Δ) | 2,26 | C6 transcription factor |  | 4 | A | 0 |
| Pa_5_7750 | 2,25 | polyketide synthase |  | 4 | A | 0 |
| Pa_1_10600 | 2,25 | mitochondrial deoxynucleotide carrier |  | 4 | A | 0 |
| Pa_1_12800 | 2,24 | unknown function |  | 4 | A | 0 |
| Pa_7_950 | 2,24 | unknown function |  | 8 | 0 | 0 |
| Pa_1_8280 | 2,23 | unknown function |  | 8 | 0 | 0 |
| Pa_1_15470 | 2,23 | laccase |  | 4 | A | 0 |
| Pa_3_1990 | 2,22 | polyketide synthase |  | 8 | 0 | 0 |
| Pa_2_3690 (Δ) | 2,22 | unknown function |  | 2 | A | R |
| Pa_0_1190 | 2,2 | molybdenum cofactor sulfurase |  | 6 | 0 | A |
| Pa_2_10220 | 2,19 | Glutamine synthetase |  | 4 | A | 0 |
| Pa_7_3250 | 2,18 | unknown function |  | 4 | A | 0 |
| Pa_4_5450 (Δ) | 2,17 | unknown function |  | 2 | A | R |
| Pa_5_3130 | 2,16 | esterase/lipase |  | 4 | A | 0 |
| Pa_4_9520 (Δ) | 2,16 | copper fist DNA binding domain protein |  | 4 | A | 0 |
| Pa_1_1230 | 2,14 | unknown function |  | 8 | 0 | 0 |
| Pa_4_3860 | 2,09 | esterase/lipase |  | 8 | A | 0 |
| Pa_2_1170 (Δ) | 2,09 | unknown function |  | 4 | A | 0 |
| Pa_2_11120 | 2,08 | malic enzyme |  | 8 | A | 0 |
| Pa_1_15590 | 2,08 | unknown function |  | 4 | A | 0 |
| Pa_6_3770 (Δ) | 2,06 | C6 transcription factor |  | 4 | A | 0 |
| Pa_1_15330 | 2,06 | membrane transport protein |  | 4 | A | 0 |
| Pa_2_50 | 2,05 | mannose-6-phosphate isomerase |  | 4 | A | 0 |
| Pa_1_20510 | 2,05 | sugar transporter | + | 2 | A | R |
| Pa_4_230 | 2,04 | unknown function |  | 1 | 0 | 0 |
| Pa_5_570 | 2,04 | unknown function |  | 8 | 0 | 0 |
| Pa_1_22300 | 2,03 | glycine dehydrogenase |  | 8 | A | 0 |
| Pa_4_3210 | 2,03 | unknown function |  | 4 | A | 0 |
| Pa_2_7590 | 2,02 | unknown function | + | 8 | 0 | 0 |
| Pa_3_720 | 2,01 | unknown function |  | 4 | A | 0 |
| Pa_2_5340 | 2,01 | esterase/lipase |  | 4 | A | 0 |

^a^ Genes selected for deletion are marked with Δ.

^b^ A: gene induced by FPR1; R: gene repressed by FPR1; 0: gene not controlled by FPR1.

^c^ A: gene induced by FMR1; R: gene repressed by FMR1; 0: gene not controlled by FMR1.
