# Supplementary material for: Genome-Wide Gene Expression Profiling of Fertilization Competent Mycelium in Opposite Mating Types in the Heterothallic Fungus Podospora anserina
Source: PLoS One. 2011 Jun 28;6(6):e21476. doi: 10.1371/journal.pone.0021476 (PMC3125171; doi:10.1371/journal.pone.0021476)
Supplement: Table S4 — Main features of genes accumulating transcripts in the mat− strains. (DOC) [file pone.0021476.s004.doc]

**Table S4.** Main features of genes accumulating transcripts in the *mat-* strain.

| Gene number^a^ | FC | Gene name or function | mat- specific expression | Class | FPR1^b^ | FMR1^c^ |
| --- | --- | --- | --- | --- | --- | --- |
| Pa_1_8290 | -51,54 | *MFM* |  | 5 | 0 | A |
| FMR1 | -13,91 | *FMR1* |  | 1 | 0 | 0 |
| Pa_6_7350 (Δ) | -6,56 | protease |  | 2 | R | A |
| Pa_7_60 | -5,97 | unknown function |  | 5 | 0 | A |
| Pa_7_9070 (Δ) | -5,62 | *PRE1* |  | 5 | 0 | A |
| Pa_4_1290 | -5,05 | carbohydrate esterase | + | 5 | 0 | A |
| Pa_5_10930 (Δ) | -4,72 | unknown function |  | 3 | R | A |
| nad1 | -4,25 | *nad1* |  | 1 | 0 | 0 |
| Pa_5_10935 (Δ) | -4,21 | unknown function |  | 5 | 0 | A |
| Pa_6_10330 (Δ) | -3,91 | polyketide synthase | + | 5 | 0 | A |
| Pa_2_13500 | -3,72 | unknown function |  | 4 | R | 0 |
| Pa_6_8610 | -3,62 | unknown function |  | 2 | R | A |
| Pa_3_10350 | -3,61 | unknown function | + | 8 | 0 | 0 |
| Pa_0_850 | -3,45 | unknown function |  | 5 | 0 | A |
| Pa_5_10945 (Δ) | -3,27 | unknown function |  | 3 | R | A |
| Pa_7_875 (Δ) | -3,11 | unknown function |  | 5 | 0 | A |
| Pa_5_6620 | -3,08 | cytochrome P450 |  | 4 | R | 0 |
| Pa_0_1140 | -3,05 | hydroxyisobutyrate dehydrogenase |  | 5 | 0 | A |
| nad3 | -3 | *nad3* |  | 1 | 0 | 0 |
| Pa_1_1310 | -2,93 | unknown function |  | 4 | R | 0 |
| Pa_6_6400 | -2,89 | glycoside hydrolase |  | 5 | 0 | A |
| Pa_7_4140 | -2,88 | gibberellin dioxygenase |  | 5 | 0 | A |
| Pa_5_6640 | -2,85 | cytochrome P450 |  | 2 | R | A |
| Pa_2_1200 | -2,81 | agmatinase 1 precursor |  | 3 | R | A |
| Pa_1_21320 | -2,69 | unknown function |  | 4 | R | 0 |
| Pa_3_7460 | -2,67 | alcohol dehydrogenase |  | 2 | R | A |
| Pa_1_21830 | -2,66 | biphenyl dioxygenase |  | 4 | R | 0 |
| Pa_4_1433 | -2,64 | unknown function | + | 8 | 0 | 0 |
| Pa_5_7120 | -2,62 | unknown function |  | 4 | R | 0 |
| Pa_1_6014 | -2,62 | unknown function | + | 1 | 0 | 0 |
| Pa_5_10940 (Δ) | -2,61 | unknown function |  | 5 | 0 | A |
| Pa_1_21970 | -2,6 | peroxisomal alcohol oxidase |  | 8 | 0 | A |
| Pa_1_21840 | -2,55 | unknown function |  | 4 | R | 0 |
| nad2 | -2,53 | *nad2* |  | 1 | 0 | 0 |
| Pa_5_2930 | -2,52 | glucose transporter |  | 4 | R | 0 |
| Pa_2_80 | -2,52 | transporter protein |  | 8 | 0 | A |
| Pa_5_1230 | -2,49 | unknown function |  | 5 | 0 | A |
| Pa_7_15 | -2,46 | unknown function |  | 8 | 0 | 0 |
| Pa_1_5700 | -2,4 | potassium transporter |  | 8 | R | 0 |
| Pa_1_14680 | -2,37 | 3-ketoacyl-(acyl-carrier-protein) reductase |  | 4 | R | 0 |
| Pa_5_10190 | -2,36 | glycosyl transferase |  | 4 | R | 0 |
| Pa_3_830 | -2,25 | carbohydrate esterase |  | 8 | 0 | 0 |
| Pa_5_1890 | -2,24 | unknown function |  | 4 | R | 0 |
| Pa_2_14160 | -2,2 | lactose permease |  | 5 | 0 | A |
| Pa_6_6730 | -2,17 | unknown function |  | 8 | R | A |
| Pa_1_3100 | -2,16 | unknown function |  | 8 | 0 | A |
| Pa_2_8150 | -2,15 | unknown function |  | 8 | 0 | A |
| Pa_4_4560 | -2,15 | zeaxanthin epoxidase |  | 8 | R | A |
| Pa_1_1490 | -2,13 | sorbitol dehydrogenase |  | 8 | R | 0 |
| Pa_6_11500 | -2,13 | polysaccharide lyase |  | 8 | 0 | 0 |
| Pa_7_140 | -2,12 | uracil phosphoribosyltransferase |  | 8 | 0 | 0 |
| Pa_1_13200 | -2,12 | unknown function |  | 8 | 0 | 0 |
| Pa_3_130 | -2,11 | unknown function |  | 8 | R | 0 |
| Pa_5_1620 | -2,1 | NADH oxidase |  | 5 | 0 | R |
| Pa_2_4220 | -2,09 | unknown function |  | 4 | R | 0 |
| Pa_4_9880 | -2,09 | unknown function |  | 4 | R | 0 |
| Pa_4_5280 | -2,09 | unknown function |  | 8 | R | 0 |
| Pa_1_11070 | -2,08 | glycoside hydrolase |  | 8 | 0 | 0 |
| Pa_2_13480 | -2,08 | unknown function |  | 8 | 0 | 0 |
| Pa_3_10430 | -2,08 | unknown function | + | 8 | 0 | 0 |
| Pa_5_7010 | -2,07 | unknown function |  | 8 | 0 | 0 |
| Pa_4_9550 | -2,07 | unknown function |  | 8 | 0 | A |
| Pa_3_5600 | -2,05 | unknown function |  | 8 | 0 | 0 |
| Pa_2_13420 | -2,05 | unknown function |  | 8 | 0 | 0 |
| Pa_1_17920 | -2,03 | phosphate transporter |  | 5 | 0 | A |
| Pa_6_10 | -2,02 | unknown function |  | 4 | R | 0 |
| Pa_0_1080 | -2,01 | unknown function |  | 8 | 0 | 0 |
| Pa_4_110 (Δ) | -2 | unknown function |  | 5 | 0 | A |
| Pa_5_1550 | -2 | unknown function |  | 8 | R | 0 |

^a^ Genes selected for deletion are marked with Δ.

^b^ A: gene activated by FPR1; R: gene repressed by FPR1; 0: gene not controlled by FPR1.

^c^ A: gene activated by FMR1; R: gene repressed by FMR1; 0: gene not controlled by FMR1.
