# Supplementary material for: Genome-Wide Gene Expression Profiling of Fertilization Competent Mycelium in Opposite Mating Types in the Heterothallic Fungus Podospora anserina
Source: PLoS One. 2011 Jun 28;6(6):e21476. doi: 10.1371/journal.pone.0021476 (PMC3125171; doi:10.1371/journal.pone.0021476)
Supplement: Table S5 — RT-qPCR validation of microarray data. Cq values are in Table S6. (DOC) [file pone.0021476.s005.doc]

**Table S5.** RT-qPCR validation of microarray data. Cq values are in Table S6.

| Gene number | Gene name or function | Microarray fold change | qPCR fold change | Std. Error | 95% C.I. | p-value | Result |
| --- | --- | --- | --- | --- | --- | --- | --- |
| Pa_1_8290 | MFM | -51.5 | -6,925 | -1,699 to  -19,660 | -1,319 to -28,983 | 0.003 | DOWN |
| Pa_7_9070 | PRE1 | -5.6 | -220 | -156 to -283 | -130 to -385 | 0 | DOWN |
| Pa_6_7350 | protease | -6.6 | -5.4 | -4.1 to -6.9 | -3.7 to -8.3 | 0 | DOWN |
| Pa_5_2930 | Glc_trans | -2,5 | 1.05 | 1.4 to -1.2 | 1.6 to -1.2 | 0.75 | NS^c^ |
| Pa_5_6620 | P450 | -3 | -1.1 | 1.2 to -1.5 | 1.4 to -1.6 | 0.32 | NS^c^ |
| Pa_2_2310 | MFP | 144 | 6,242 | 548 to 45,000 | 381 to 51,500 | 0.003 | UP |
| Pa_4_3858 | 3858 | 48 | 65 | 42 to 97 | 34 to 129 | 0.008 | UP |
| Pa_1_24410 | SAM | 28 | 115 | 88 to 152 | 81 to 166 | 0.007 | UP |
| Pa_4_1380 | PRE2 | 11 | 77 | 60 to 86 | 57 to 90 | 0.003 | UP |
| Pa_5_9770 | PAG | 6 | 440 | 240 to 914 | 147 to 1,410 | 0.007 | UP |
| Pa_3_1710 | AOX | 4.6 | 2.2 | 1.8 to 2.9 | 1.3 to 3.9 | 0.007 | UP |
| Pa_4_3160 | PEPCK | 3.5 | 1.2 | 1.0 to 1.4 | -1.04 to 1.6 | 0.025 | UP |
| Pa_2_5340 | lipase | 2 | 1.2 | -1.2 to 1.6 | -1.3 to 2.0 | 0.311 | NS^c^ |
| Pa_4_80^a^ | Methyl-transferase | 2.4 | 5.3 | 3.9 to 6.7 | 3.3 to 8 | 0.008 | UP |
| Pa_4_80^b^ | Methyl-transferase | 2.4 | 4.6 | 3.3 to 5.9 | 3.0 to 6.3 | 0,007 | UP |

^a^ RT-qPCR primers were designed in the 3’ part of the coding sequence.

^b^ RT-qPCR primers were designed in the 5’ part of the coding sequence.

^c^ not significant p-value.
