# Supplementary material for: Genome-Wide Gene Expression Profiling of Fertilization Competent Mycelium in Opposite Mating Types in the Heterothallic Fungus Podospora anserina
Source: PLoS One. 2011 Jun 28;6(6):e21476. doi: 10.1371/journal.pone.0021476 (PMC3125171; doi:10.1371/journal.pone.0021476)
Supplement: Table S13 — Oligonucleotide primers used for RT-qPCR. (DOC) [file pone.0021476.s013.doc]

**Table S13.** Oligonucleotide primers used for RT-qPCR.

| Type of gene^a^ | Gene number | Gene name or function | Primer name | Primer sequence 5’>3’ ^b^ | Amplicon size |
| --- | --- | --- | --- | --- | --- |
| HKG | Pa_1_16650 | AS1 | AS1f | CAACATGGCTGACGAATAC/AACGC | 115 bp |
|  |  |  | AS1r | GGAGGTCAGGTCAAGGAGA/GCATC |  |
| HKG | Pa_3_6780 | CIT1 | CIT1f | CTCCTCCAAGACCCAG/ACCCTC | 100 bp |
|  |  |  | CIT1r | GACCTTGGAGCCATGCTCC/TTTC |  |
| HKG | Pa_3_5110 | GPD | GPDf | CATTGAGCCCAAGTACGCT/GAG | 113 bp |
|  |  |  | GPDr | GTCGCGCTCAGTGTAGAACTTGA |  |
| HKG | Pa_5_5390 | H2A | H2Af | GCAAGAACGCGCAATC/TCGTTC | 329 bp |
|  |  |  | H2Ar | AGTCTTCTTGGGAAGAAGGT/TCTG |  |
| HKG | Pa_7_6690 | PDF2 | PDFf | GCAGACAGGTTCGAAAAG/ATTG | 294 bp |
|  |  |  | PDFr | CAGATGATCAATGGTT/TCTTGC |  |
| HKG | Pa_4_8980 | TBP | TBPf | CACACCCACTCTTCA/GAACATT | 106 bp |
|  |  |  | TBPr | ACGCTTGGGGTTGTA/CTCAGC |  |
| HKG | Pa_7_8490 | TIP41 | TIPf | GTTTGCGGAGGTGAAGAAG/AA | 146 bp |
|  |  |  | TIPr | CCGTCTCACCCTCGAGAC |  |
| HKG | Pa_4_7790 | UBC | UBCf | GGCCATCCCCATCCATCAAC | 107 bp |
|  |  |  | UBCr | GGTGATGGTCTTGCCAGTGA/GA |  |
| GoI | N/A^c^ | FMR1 | FMR1f | GGTTTCATGGGCTACCGAT/CCTAC | 251 bp |
|  |  |  | FMR1r | CATCCAAGGGCTTCCATGTAGC |  |
| GoI | N/A^c^ | SMR1 | SMR1f | ATTCAGGCAACTGATGCTAGAA/CAT | 186 bp |
|  |  |  | SMR1r | GTGTATTCAGGACGGGGTAT/CATA |  |
| GoI | N/A^c^ | SMR2 | SMR2f | CGCTGCCACCAACTTCAG/CAAAA | 162 bp |
|  |  |  | SMR2r | ACTAGATCGCCAATAGTCA/CAGTC |  |
| GoI | Pa_1_20590 | FPR1 | FPR1f | GGCGTTCTCAATACAATGAA/GTCG | 297bp |
|  |  |  | FPR1r | GCCACCAGTCATGACGGAAATG |  |
| GoI | Pa_1_8290 | MFM | MFMf | CCACCCTCGCAACAACACGTTAGA | 150 bp |
|  |  |  | MFMr | AAACGAAGGCGATGCTCATGTTGG |  |
| GoI | Pa_7_9070 | PRE1 | 9070f | CGGCGGTCATCTTTACGGT | 195 bp |
|  |  |  | 9070r | GGTAAAAGGTGAGGC/AAGCC |  |
| GoI | Pa_6_7350 | protease | 7350f | AAGTTCCCTGCTCGATGGT | 205 bp |
|  |  |  | 7350r | GTTTGGTCAAACACGA/CATAC |  |
| GoI | Pa_5_2930 | Glc-trans | 2930f | GGAAAGGGGTGGAGGAAGT | 228 bp |
|  |  |  | 2930r | CTTGTCAATGAAAAGC/CACATG |  |
| GoI | Pa_5_6620 | P450 | 6620f | AAGTACGGACTTTTGCGCTGGTT | 234 bp |
|  |  |  | 6620r | CGACCGAATCGTGCT/TGCC |  |
| GoI | Pa_2_2310 | MFP | MFPf | CGTACGGGAGTGGACTTGGATGGA | 149 bp |
|  |  |  | MFPr | CGACACTGAGGCGGTACCCAAAAG |  |
| GoI | Pa_4_3858 | 3858 | 3858f | CTTTCCTGACGATTC/GACC | 171 bp |
|  |  |  | 3858r | CAAGTGTATTGCTTGTGGGTT |  |
| GoI | Pa_1_24410 | SAM | 24410f | GCACCATCACCAAAGCAATC | 151 bp |
|  |  |  | 24410r | TCTTCAAAGGGGAGTTT/CACA |  |
| GoI | Pa_4_1380 | PRE2 | 1380f | GTTGATGTTTGTGCCCG/TGG | 177 bp |
|  |  |  | 1380r | TCGGTGGTTGTGCCAGTCG |  |
| GoI | Pa_5_9770 | PAG | 9770f | ATGAAGCGCAGCAGGATGT | 147 bp |
|  |  |  | 9770r | TGTCAAAGATGACTTTG/AATCTC |  |
| GoI | Pa_3_1710 | AOX | 1710f | CTGGTCCGTCTGTGTCG/ATGG | 139 bp |
|  |  |  | 1710r | AAATGAACCGAACAAG/CCATT |  |
| GoI | Pa_4_3160 | PEPCK | 3160f | CCGAGAACGAAATCTG/GTGG | 196 bp |
|  |  |  | 3160r | ATGAAGAGGGCATGGTAGGC |  |
| GoI | Pa_2_5340 | lipase | 5340f | TGATCGGCCACTCCAT/GGG | 226 bp |
|  |  |  | 5340r | CGGATTGTGACGGAGGGTTC |  |
| GoI | Pa_4_80 | Methyl transferase1 | 80f_I1 | GCTTACATTATGCTCTT/TGGC | 209 bp |
|  |  |  | 80r_I1 | CCGTTGGTTCCGTTCTCAC |  |
| GoI | Pa_4_80 | Methyl transferase4 | 80f_I4 | GCCGAATTCTCATCTG/CGA | 243 bp |
|  |  |  | 80r_I4 | CCAAACCAACCTGGCTTCT |  |

^a^: HKG: housekeeping gene, used as candidate for reference gene; GoI: gene of interest.

^b^: slashes (/) separate sequences on two consecutive exons.

^c^: not applicable
